# Loop closure detection of visual SLAM based on variational autoencoder

**DOI:** 10.3389/fnbot.2023.1301785

**Published:** 2024-01-19

**Authors:** Shibin Song, Fengjie Yu, Xiaojie Jiang, Jie Zhu, Weihao Cheng, Xiao Fang

**Affiliations:** ^1^Department of College of Electrical Engineering and Automation, Shandong University of Science and Technology, Qingdao, China; ^2^Yantai Tulan Electronic Technology Co., Ltd, Yantai, China

**Keywords:** visual SLAM, loop closure detection, variational autoencoder, attention mechanism, loss function

## Abstract

Loop closure detection is an important module for simultaneous localization and mapping (SLAM). Correct detection of loops can reduce the cumulative drift in positioning. Because traditional detection methods rely on handicraft features, false positive detections can occur when the environment changes, resulting in incorrect estimates and an inability to obtain accurate maps. In this research paper, a loop closure detection method based on a variational autoencoder (VAE) is proposed. It is intended to be used as a feature extractor to extract image features through neural networks to replace the handicraft features used in traditional methods. This method extracts a low-dimensional vector as the representation of the image. At the same time, the attention mechanism is added to the network and constraints are added to improve the loss function for better image representation. In the back-end feature matching process, geometric checking is used to filter out the wrong matching for the false positive problem. Finally, through numerical experiments, the proposed method is demonstrated to have a better precision-recall curve than the traditional method of the bag-of-words model and other deep learning methods and is highly robust to environmental changes. In addition, experiments on datasets from three different scenarios also demonstrate that the method can be applied in real-world scenarios and that it has a good performance.

## 1 Introduction

Loop closure detection is the process of identifying the places that a robot has visited before, which can help the robot relocate when it loses its trajectory due to motion blur, forming a topologically consistent trajectory map (Gálvez-López and Tardis, 2012; Arshad and Kim, 2021). The key to solving the loop closure detection problem is to match the images captured by the robot with the images corresponding to previously visited locations on the map. Loop closure detection is essentially an image-matching problem, the core of which is the representation and matching of image features.

Traditional loop closure detection methods are generally based on appearance (Cummins and Newman, 2008), which has little connection with the front end and back end of the system. The loop detection relationship is only determined given the similarity of the two images. Since proposed, the appearance-based loop closure detection methods have become mainstream in visual SLAM and have been applied to practical systems (Mur-Artal et al., 2015), most of which utilize the bag-of-words (BOW) model (Filliat, 2007; Garcia-Fidalgo and Ortiz, 2018; Li et al., 2019). The BOW model clusters the visual feature descriptors of the image to build a dictionary and then searches the words that match the features of each image to describe the image. A word can be regarded as a representative of several similar features.

However, appearance-based methods usually depend on traditional handcrafted features, such as SIFT (Lowe, 2004), SURF (Bay et al., 2006), and BRIEF (Calonder et al., 2010). Each of these features has its own characteristics, but they have limited ability to express the environment under the conditions of significant changes in viewing angle or illumination conditions. Moreover, they can only describe the local appearance, which has a limited ability to describe the whole image. BOW-based closed-loop detection methods rely on appearance features and their presence in the dictionary, ignoring geometric information and relative positions in space, thus generating false loops due to similar features appearing in different places (Qin et al., 2018; Arshad and Kim, 2021).

Recently, given the rapid development of deep learning in computer vision (Bengio et al., 2013), methods based on convolutional neural networks (CNN) (Farrukh et al., 2022; Favorskaya, 2023) and attention mechanisms have attracted more attention in imitating human cognitive patterns. Using the learning features of the neural network to replace the traditional manual features is a new method to solve the loop detection problem (Memon et al., 2020; Wang et al., 2020). Zhang et al. (2022) used global features to perform candidate frame selection via HNSW (Malkov and Yashunin, 2018), while the local one was exploited for geometric verification via LMSC. Based on the above two components, the whole system was at the same time high-performance and efficient compared with state-of-the-art approaches. Liu and Cao (2023) utilized the effective FGConv (Liu et al., 2020) as their proposed network backbone due to its high efficiency. The network adopts an encoder-decoder-based structure with skip connections. Osman et al. (2023) trained PlaceNet to identify dynamic objects in scenes via learning a grayscale semantic map indicating the position of static and moving objects in the image. PlaceNet is a multi-scale deep autoencoder network augmented with a semantic fusion layer for scene understanding, which generates semantic-aware deep features that are robust to dynamic environments and scale invariance.

At the same time, the attention mechanism can weigh key information and ignore other unnecessary information to process information with higher accuracy and speed. Hou et al. (2015) used the pre-trained CNN model to extract features to obtain a complete image representation, and through experiments on various datasets, it was shown that CNN features are more robust to changes in visual angle, light intensity, and scale of the environment. Gao and Zhang (2017) used a modified stacked denoising autoencoder (SDA), a deep neural network trained in an unsupervised manner, to solve the loop closure detection problem, but the extraction speed is slow. NetVLAD (Arandjelovic et al., 2016) is currently an advanced location recognition method, which is an improved version of VLAD. It clusters local descriptors into global image descriptors through neural network learning, which has high accuracy and applicability. Schönberger et al. (2018) used a variational autoencoder (VAE) to compress and encode 3D geometric and semantic information to generate a descriptor for subsequent position recognition. This method has good detection accuracy for large viewing angles and appearance changes, but its computational cost is high.

For traditional methods, the problem of false detection is easy to occur when facing similar environments or relatively large changes in illumination, which leads to serious errors in map estimation. In this research paper, we propose a loop closure detection method based on a variational autoencoder to solve the loop closure detection problem in visual SLAM. The method uses intermediate layer depth features instead of the traditional manual features and compares the current image with the previous keyframes to detect the loop. The method incorporates an attentional mechanism in the neural network to obtain more useful features and also improves the loss function of the network and eliminates erroneous loops through geometric consistency.

## 2 Loop closure detection system architecture based on variational autoencoder

**Figure 1:**
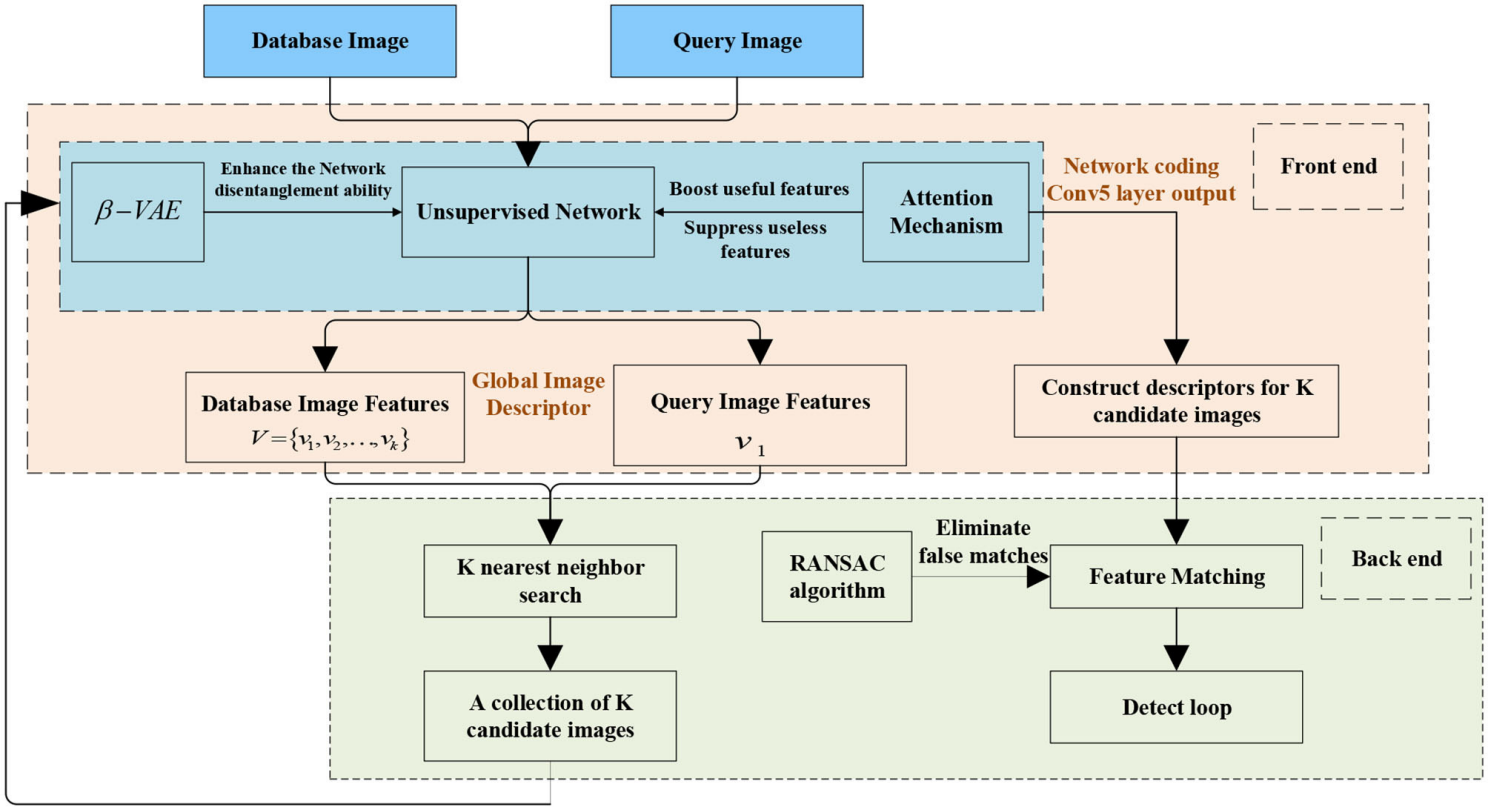
Function diagram of loop detection based on a variational autoencoder.

Figure 1 shows the structure of the proposed loop closure detection system based on a variational autoencoder. Dividing loop closure detection into two parts: front-end feature extraction and back-end feature matching. The proposed method consists of two sections: (1) In the front-end feature extraction part, a network structure based on a variational autoencoder is designed and constructed, and the attention mechanism is added. It will be called SENet-VAE. Besides, the loss function of the variational autoencoder is revised and improved. The aim is to learn feature representations with fewer image features to obtain more accurate results. (2) In the back-end feature matching part, due to the low dimensionality of the descriptor, a K-nearest neighbor search is used to detect loop closures and geometric checks are used to filter false detections.

**Figure 2:**
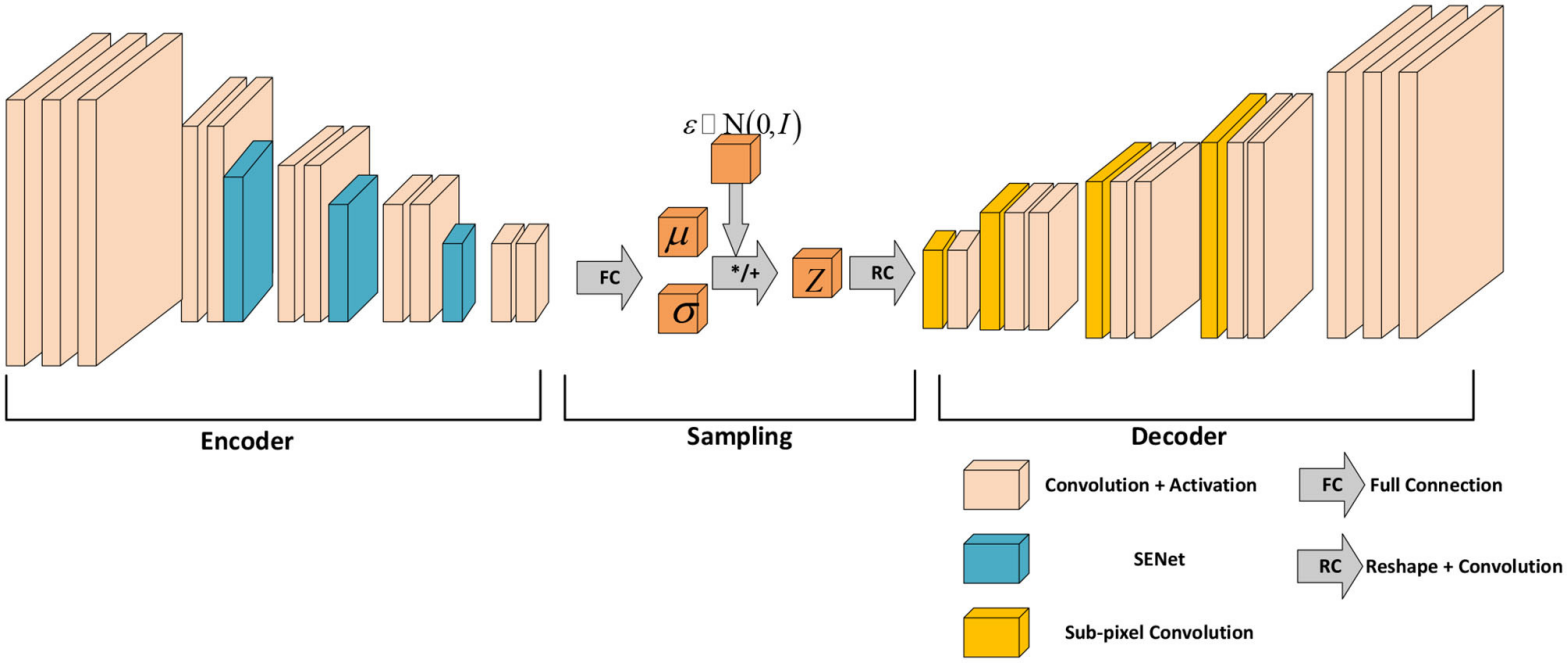
Feature extraction network structure of SENet-VAE.

The proposed network structure is shown in Figure 2. The front encoder part encodes the input image with 13 convolutional layers, four pooling layers, and three SENet attention modules. The middle section is responsible for sampling and mapping the encoder input to a normal distribution. The later decoder part performs the semantic segmentation of the image and the decoded reconstruction of the image with eight convolutional layers and four upsampling layers. The last decoder outputs high-dimensional features to the softmax classifier. The classifier classifies the pixels of the input image and predicts the probability of the classification labels while decoding the image.

The proposed network is based on VAE. The network input is an RGB image with a resolution of 192 × 256. The encoder maps the image to a normal distribution through the latent variables μ and σ, and then the information of the potential variables is decoded by the decoder. It describes the observation of the potential space in a probabilistic way. In addition, this method adds an attentional mechanism to the VAE to increase the weight of the effective features to obtain better results.

### 2.1 Improvement of the objective function of the VAE network

Inspired by Sikka et al. (2019), the loss function of VAE is improved based on the KL divergence of traditional VAE. A hyperparameter β is added to the second KL divergence of the loss function. As the parameter β rises, the traditional VAE has the characteristics of disentanglement. The entangled data in the original data space are transformed into a better representation space, in which the changes of different elements can be separated from each other.

Assume that the network input data *D* = {*X, V, W*} is a set composed of images *x*, conditional independent factors *v*, and conditional correlation factors *w*. Suppose that the conditional probability distribution of *x*, denoted by *p*(*x*|*v, w*), is generated from simulated real data consisting of *v* and *w*, which is shown in Equation (1):


(1) $$\begin{array}{l} p\left(x|v,w\right)=Sim\left(v,w\right) \end{array}$$

where *Sim*() is the simulation operation.

It is hoped that the generative model will learn a model *p*(*x*|*z*) that can generate pictures through a hidden layer *z* and make this generative process as close as possible to real-world models. The mathematical expression is shown in Equation 2.


(2) $$\begin{array}{l} p\left(x|z\right)\approx p\left(x|v,w\right)=Sim\left(v,w\right) \end{array}$$

This model is controlled by the parameter θ. Therefore, an appropriate goal is to maximize the marginal likelihood of the observed data *x* in the expectation over the entire distribution of the latent factor *z*. Which is shown in Equations (3) and (4).


(3) $$\begin{array}{l} p_{\theta }\left(x\right)=\underset{z}{\sum }p_{\theta }\left(z\right)p_{\theta }\left(x|z\right)=E_{p_{\theta }\left(z\right)}\left[p_{\theta }\left(x|z\right)\right] \end{array}$$

(4) $$\begin{array}{l} \underset{\theta }{\max}E_{p_{\theta }\left(z\right)}\left[p_{\theta }\left(x|z\right)\right]. \end{array}$$

For *p*(*z*), as its definite form cannot be determined, it is often approximated by a joint distribution model *q*_ϕ_(*z*|*x*). In order for *q*_ϕ_(*z*|*x*) to be as simple as possible, it is approximated by a Gaussian distribution *p*(*z*) ~ *N*(0, *I*), as follows in Equation (5).


(5) $$\begin{array}{l} \underset{\phi ,\theta }{\max}E_{x\sim D}\left[E_{q_{\phi }\left(z|x\right)}\left[\log p_{\theta }\left(x|z\right)\right]\right]s.t.D_{KL}\left(q_{\phi }\left(z|x\right)\|p\left(z\right)\right)\leq \varepsilon \end{array}$$

Rewrite the above equation as the Lagrange equation under the Karush-Kuhn-Tucker (KKT) condition:


(6) $$\begin{array}{ll} F\left(\theta ,\phi ,\beta ;x,z\right)=E_{q_{\phi }\left(z|x\right)}\left[\log p_{\theta }\left(x|z\right)\right]\\ - & \beta \left(D_{KL}\left(q_{\phi }\left(z|x\right)\|p\left(z\right)\right)-\varepsilon \right) \end{array}$$

Since β, ε ≥ 0, according to the complementary relaxation degree KKT condition, Equation (6) can be rewritten to obtain the β − *VAE* formula as the ultimate objective function, as follows in Equation (7):


(7) $$\begin{array}{l} L_{\beta -KLD}=F\left(\theta ,\phi ,\beta ;x,z\right)\geq L\left(\theta ,\phi ;x,z,\beta \right)\\ =E_{q_{\phi }\left(z|x\right)}\left[\log p_{\theta }\left(x|z\right)\right]-\beta D_{KL}\left(q_{\phi }\left(z|x\right)\|p\left(z\right)\right) \end{array}$$

As the value β becomes larger, *q*_ϕ_(*z*|*x*) becomes simpler, transmitting less information and still being able to reconstruct the image well.

After sampling from the standard normal distribution ε, the latent variable *z* obtained by the encoder is sent to the decoder, which is used to predict the full-resolution semantic segmentation label and to reconstruct the full-resolution RGB image. The output of the decoder is then used to construct the RGB reconstruction loss function *L*_*r*_, as follows in Equation (8) and the maximum cross-entropy loss function *L*_*s*_ to account for class bias, as follows in Equation (9):


(8) $$\begin{array}{l} L_{r}=-\underset{i}{\sum }\left(x_{i}\log\left(p_{i}\right)+\left(1-x_{i}\right)\log\left(1-p_{i}\right)\right) \end{array}$$

(9) $$\begin{array}{l} L_{s}=\frac{1}{N}\underset{i}{\sum }L_{i}=\frac{1}{N}\underset{i}{\sum }\overset{M}{\underset{c=1}{\sum }}y_{ic}\log\left(p_{ic}\right) \end{array}$$

Here *x*_*i*_ and *p*_*i*_ represent the label of the input image and the probability of the positive class output by the network behind the softmax function, respectively. M represents the number of categories, *y*_*ic*_ is the sign function (0 or 1), and *p*_*ic*_ is the probability that the observation sample i belongs to category c, which is obtained by the softmax function.

In the encoder part, the weight of the two encoders is shared in the form of a triple network, and a sample is selected from the dataset called anchor. Samples of the same type as the anchor are selected. Distortion or darkening operations are performed, and the movement of the camera is imitated to a certain extent. This type of image is called a positive image. In the data of the current training batch, the sample that is different from the anchor is called a negative image. Anchor, positive image, and negative image consist of a triplet. The global image descriptor is taken from the latent variable μ. With the descriptors of a baseline image *d*_*a*_, a positive image *d*_*p*_, and a negative image *d*_*m*_, the triplet loss function is defined as follows in Equation (10):


(10) $$\begin{array}{l} L_{t}=\max\left(0,d_{a}^{T}\left(d_{n}-d_{p}\right)+m\right) \end{array}$$

where *m* is the marginal hyperparameter.

This loss function expressed by *L*_*t*_ forces the network to learn to use m to distinguish the similarity between positive and negative images. The minimization of the damage function is obtained by minimizing the cosine similarity between the reference image and the negative image and maximizing the similarity between the reference image and the positive image.

Finally, the overall objective function is defined as follows in Equation (11):


(11) $$\begin{array}{l} L=\lambda_{0}L_{\beta -KLD}+\lambda_{1}L_{r}+\lambda_{2}L_{s}+\lambda_{3}L_{t} \end{array}$$

where λ_*i*_ is the weight factor to balance the impact of each project.

### 2.2 Attention mechanism module

The attention mechanism squeeze-and-excitation networks (SENet) (Hu et al., 2018) considers the relationship between feature channels to improve the performance of the network. The attention mechanism adopts a brand-new feature recalibration strategy, which automatically acquires the importance of each feature channel through learning. Then, useful features are promoted and features that are not very useful for the current task are suppressed based on feature weight.

The SENet module in this article changes the input from the previous pooling layer: *F*_*tr*_:*X* → *U*, *X* ∈ ℝ^*H*^′^×*W*^′^×*C*^′^^, *U* ∈ ℝ^*H*×*W*×*C*^ and transmits it to the next layer. Then, the output can be written as follows in Equation (12):


(12) $$\begin{array}{l} u_{c}=v_{c}*X=\overset{C}{\underset{s=1}{\sum }}v_{c}^{s}*x^{s} \end{array}$$

Here *F*_*tr*_ is the pooling operator, *V* = [*v*_1_, *v*_2_, …, *v*_*C*_] represents the filter, *v*_*c*_ represents the parameters of the c-th filter, *C* represents the number of channels in the feature graph, *H* represents the height of the feature graph, and *W* represents the width of the feature graph.

The goal is to ensure that the network is sensitive to its informative features so that they can be exploited subsequently and suppress useless features. Therefore, before the response enters the next transformation, it is divided into three parts, namely, squeeze, excitation, and scale, to recalibrate the filter response.

First, the squeeze operation encodes the entire spatial feature into a global feature by using global average pooling. Specifically, each two-dimensional feature channel is turned into a real number, which has a global receptive field to some extent, and the output dimension matches the number of input feature channels. It represents the global distribution of the response on the feature channel and enables the layers to be close to the input to obtain the global receptive field.

The statistic *z* ∈ ℝ^*C*^ is generated by reducing the set *U* of the local descriptor to the spatial dimension *H* × *W*, where the c-th element of *z* is calculated by Equation (13):


(13) $$\begin{array}{l} z_{c}=F_{sq}\left(u_{c}\right)=\frac{1}{H\times W}\overset{H}{\underset{i=1}{\sum }}\overset{W}{\underset{j=1}{\sum }}u_{c}\left(i,j\right) \end{array}$$

The second part is the excitation operation, which fully captures the channel dependencies by utilizing the information gathered in the squeeze operation. This part consists of two fully connected layers. The first layer is a dimension reduction layer with the parameter $W_{1}\in {\mathbb{R}}^{\frac{C}{r}\times C}$, which is activated by the ReLU activation function. The second layer is the dimensionality-increasing layer with the parameter $W_{2}\in {\mathbb{R}}^{C\times \frac{C}{r}}$, which is restored to the original dimension and uses the sigmoid activation function, as follows in Equation (14). Here, δ refers to the ReLU activation function.


(14) $$\begin{array}{l} s=F_{ex}\left(z,W\right)=\sigma \left(g\left(z,W\right)\right)=\sigma \left(W_{2}\delta \left(W_{1}z\right)\right) \end{array}$$

Finally, the scale operation part multiplies the learned activation values of each channel (sigmoid activation, value 0 to 1) by the original features on *U*, which is shown in Equation (15):


(15) $$\begin{array}{l} {\tilde{x}}_{c}=F_{scale}\left(u_{c},s_{c}\right)=s_{c}\cdot u_{c} \end{array}$$

**Figure 3:**
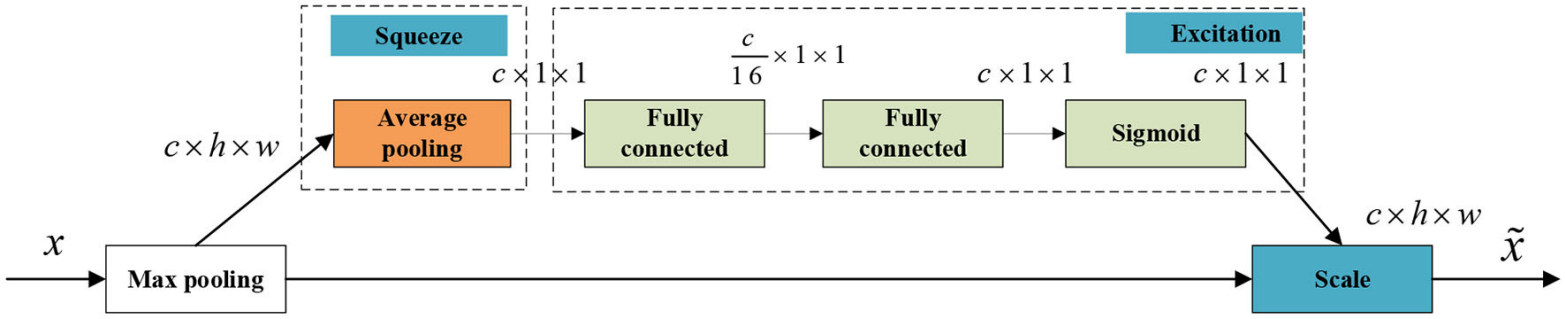
The squeeze-and-excitation block of SENet-VAE.

The construction of the squeeze-and-excitation block in the network is shown in Figure 3.

## 3 Loop closure detection based on a variational autoencoder

In this section, we use the neural network described above to extract the image features and use it to perform back-end image feature matching to achieve loop closure detection. During the image-matching process, key point mismatches are eliminated by geometric checking, which improves the accuracy of detection.

### 3.1 Image feature description

The global descriptor for the image is taken from the output of the convolutional layer where the latent variable μ is located in the sampling layer of the network. After the encoder, the latent variable *z* is split channel-wise into 14 local descriptors of size 1/4 of the input image size. One of the slices is dedicated to reconstructing the full-resolution RGB image, while the other is sent to the decoder, concatenated, and then used to predict a full-resolution semantic segmentation label. Since the local descriptor dimension is 192 dimensions, the global descriptor consisting of 14 local descriptors has a dimension of 10,752 dimensions. It can be interpreted as a set of 10,752 dimensional vectors of length *l*, with *V*^(*I*)^ denoting the corresponding output for a given input image, which is shown in Equation (16):


(16) $$\begin{array}{l} V^{\left(I\right)}=\left(v_{1}^{\left(I\right)},v_{2}^{\left(I\right)},\ldots ,v_{l}^{\left(I\right)}\right)\in {\mathbb{R}}^{l} \end{array}$$

For the extraction of image key points, the method proposed by Garg et al. (2018) is used. It extracts key points from the maximum activation area of the underlying Conv5 layer of the network encoding. The largest activation area in a 48 × 64 window is selected as a key point on the feature map. After the key points are extracted, the key point descriptor is inspired by the BRIEF (Calonder et al., 2010) descriptor. Taking the extracted key point as the center, certain point pairs are selected in a 3 × 3 size field for comparison. After all point pairs are compared, a 256-dimensional key point descriptor is obtained. During key point matching, these descriptors are directly compared using the Euclidean distance metric.

### 3.2 Loop closure detection

In order to detect loop closures, first build a database of historical image descriptors through global image descriptors. When the image to be queried is input, the global image descriptor is used to perform a K-Nearest neighbor search in the established database, and images with relatively high similarity scores are selected to form a candidate image set. Then, K candidates are screened in the candidate set through the key points described before, and the random sample consensus (RANSAC) algorithm is used to filter out false matches. The RANSACN algorithm finds an optimal homography matrix H through at least four sets of feature-matching point pairs, and the size of the matrix is 3 × 3. The optimal homography matrix H is supposed to satisfy the maximum number of matching feature points. Since the matrix is often normalized by making *h*_33_ = 1, the homography matrix, which is expressed by Equation (17), has only eight unknown parameters:


(17) $$\begin{array}{l} s\left[\begin{array}{c} x^{\prime }\\ y^{\prime }\\ 1 \end{array}\right]=\left[\begin{array}{ccc} h_{11} & h_{12} & h_{13}\\ h_{21} & h_{22} & h_{23}\\ h_{31} & h_{32} & h_{33} \end{array}\right]\left[\begin{array}{c} x\\ y\\ 1 \end{array}\right] \end{array}$$

where (*x, y*) is the corner point of the target image, (*x*′, *y*′) is the corner point of the scene image, and *s* is the scale parameter.

Then, the homography matrix is used to test other matching points under this model. Use this model to test all the data, and calculate the number of data points and projection errors that satisfy this model through the cost function. If this model is the optimal model, the corresponding cost function should obtain the minimum value. The equation for calculating the cost function *J* is as follows in Equation (18):


(18) $$\begin{array}{l} J=\overset{n}{\underset{i=1}{\sum }}{\left({x^{\prime }}_{i}-\frac{h_{11}x_{i}+h_{12}y_{i}+h_{13}}{h_{31}x_{i}+h_{32}y_{i}+h_{33}}\right)}^{2}\\ +\left({y^{\prime }}_{i}-\frac{h_{21}x_{i}+h_{22}y_{i}+h_{23}}{h_{31}x_{i}+h_{32}y_{i}+h_{33}}\right) \end{array}$$

**Figure 4:**
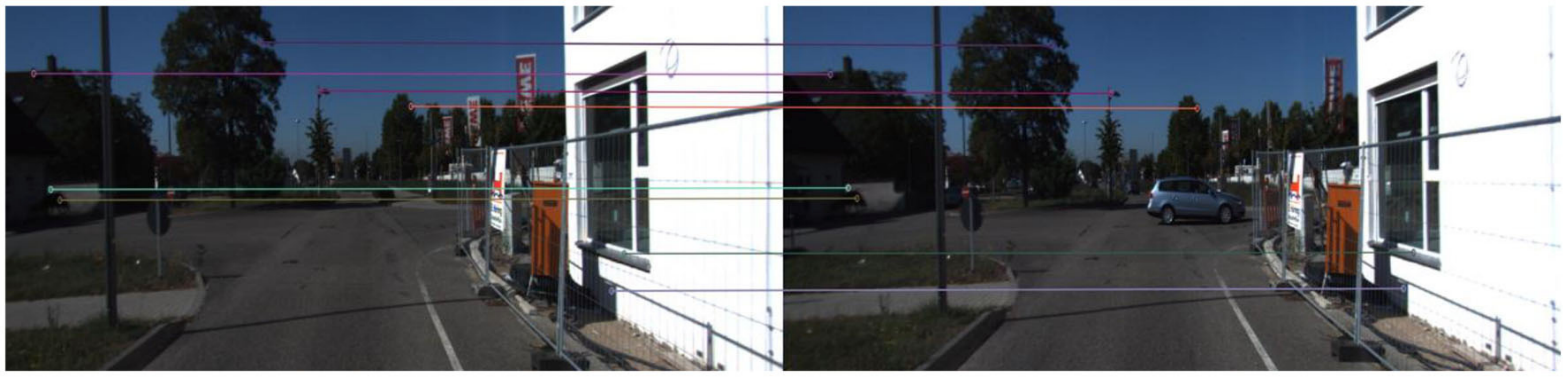
An example of final matches after performing RANSAC.

After filtering out invalid matches, the matched key points can be used to calculate the effective homography matrix as the final matching result. An example of final matches after performing RANSAC can be seen in Figure 4.

## 4 Experiments

In this section, the feasibility and performance of the proposed method will be tested on the Campus Loop dataset. The hyperparameters used in the experiments are shown in Table 1. The proposed method is compared with the BOW model and other CNN-based methods. The performance of the proposed method is measured using the precision-recall curve. There are mainly two metrics used to interpret the precision-recall curve. (1) Area under the curve (AUC), which is the area enclosed by the precision-recall curve and the coordinate axis. The closer the AUC is to 1.0, the higher the accuracy of the detection method is. (2) The maximum recall rate at 100% accuracy is represented by Max-Recall, which is the value of the recall rate when the accuracy drops from 1.0 for the first time. Finally, the KITTI odometry dataset is used to test the application and effectiveness of the proposed method in real scenarios.

**Table 1:** List of hyperparameters.

**Parameter**	**Symbol**	**Value**
Learning rate	η	10^−3^
Input image size	*I*	192 × 256
Batch size	*N*_*T*_	12
Weight function	λ_0_	10^−4^
Weight function	λ_1_	10^−4^
Weight function	λ_2_	1.0
Weight function	λ_3_	1.0
Beta parameter	β	250
Margin parameter	*m*	0.5

### 4.1 Datasets and evaluation methodology

The accuracy rate describes the probability that all the loops extracted by the algorithm are real loops, and the recall rate refers to the probability of being correctly detected in all real loops. The functions are as follows in Equations (19) and (20):


(19) $$\begin{array}{l} Precision=\frac{TP}{TP+FP} \end{array}$$

(20) $$\begin{array}{l} Recall=\frac{TP}{TP+FN} \end{array}$$

The accuracy rate and recall rate are, respectively, used as the vertical axis and horizontal axis of the precision-recall rate curve. There are four types of results for loop closure detection, as shown in Table 2. True positives and true negatives are cases where the prediction is correct. False positives are no loop closure situations that are mistaken for correct loop situations similar to potential diatheses for psychosis also known as perceptual bias (Safron et al., 2022); and false negatives are cases where a true loop situation is not detected, also known as a perceptual variance. Perceptual variance means that two images are in the same scene, but due to lighting, lens angle distortion, etc., the algorithm may misinterpret them as different scenes.

**Table 2:** Classification of loop closure detection results.

**Result/fact**	**True loop**	**False loop**
True loop	True positive (TP)	False positive (FP)
False loop	False negative (FN)	True negative (TN)

The Campus Loop dataset (Merrill and Huang, 2018) is a challenging dataset for the proposed method. The dataset consists of two sequences. These sequences are a mixture of indoor and outdoor images of the campus environment. The dataset contains large viewpoint variations, as well as illumination and appearance variations. Furthermore, each image contains different viewpoints and many dynamic objects.

On this dataset, the proposed method is compared with the following methods: (1) CNN—Zhang et al. (2017) proposed a convolutional neural network (CNN)-based loop closure detection method to input images into a pre-trained CNN model to extract features. (2) SDA—Gao and Zhang (2017) used an improved stacked denoising autoencoder (SDA) to solve the loop detection problem of the visual SLAM system. The network is trained in an unsupervised way, and the data is represented by the response of the hidden layer, which is used to compare the similarity of images. (3) DBOW—Use the DBoW2 vocabulary tree from the state-of-the-art ORB-SLAM (Mur-Artal and Tardós, 2017).

**Figure 5:**
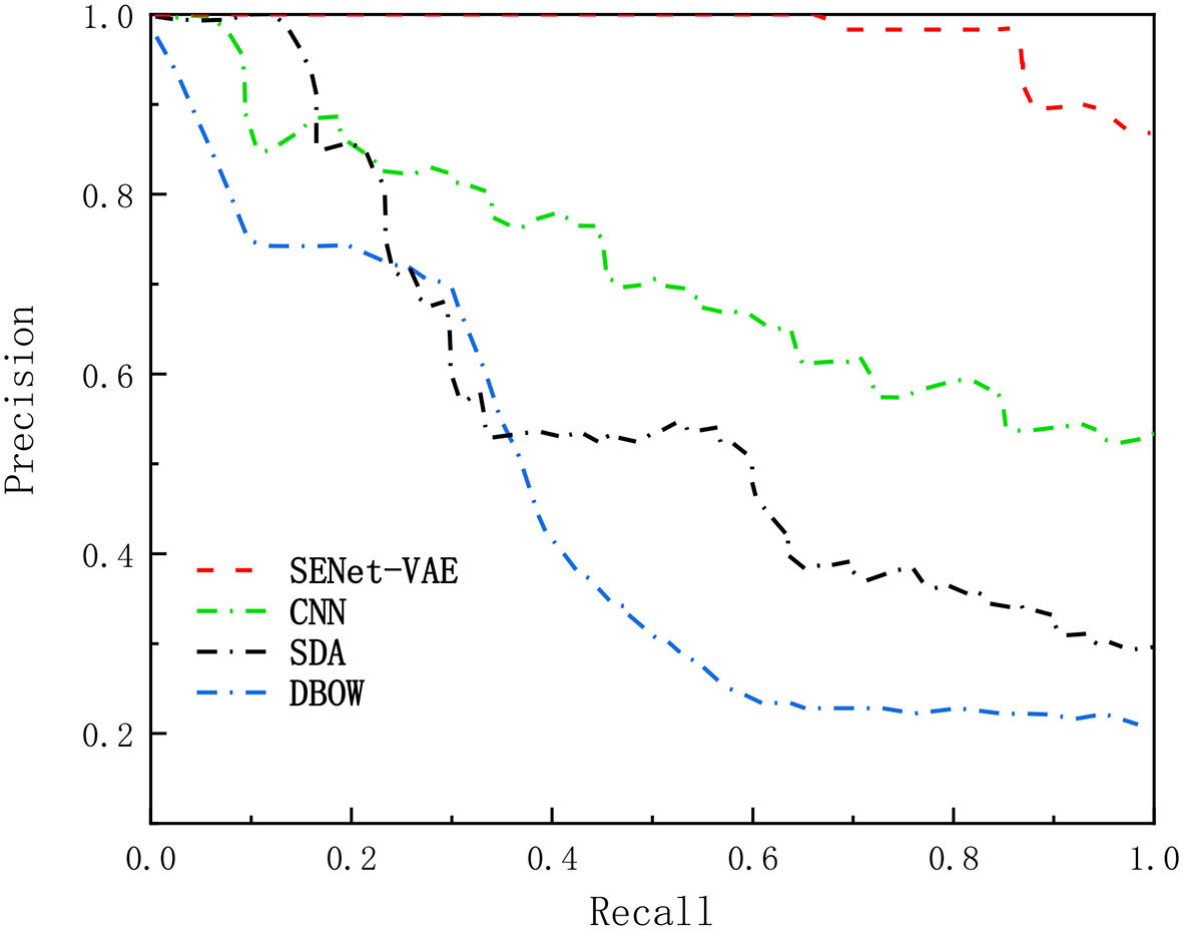
Comparison of precision-recall curves.

**Figure 6:**
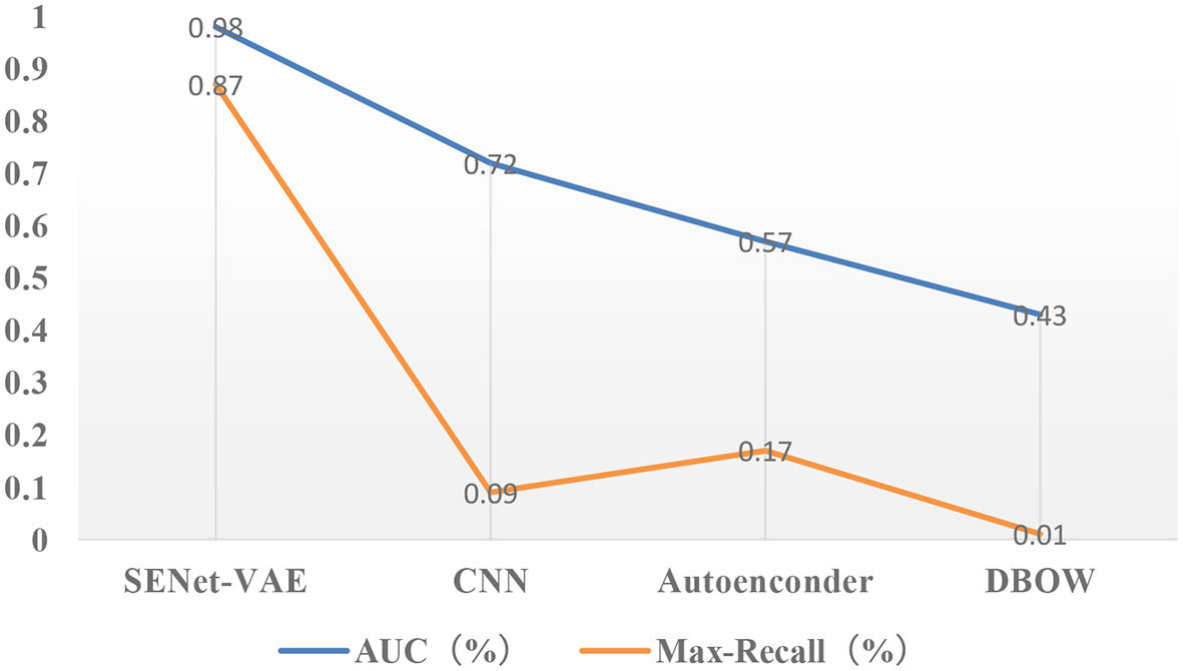
Results of precision-recall curves (the closer the AUC is to 1.0, the higher the accuracy of the detection method is; higher maximum recall means more false detections can be avoided).

Figures 5, 6 describe the results of loop closure detection on this dataset. It can be seen that the proposed method can maintain a good accuracy rate even at a high recall rate compared with other methods. Through the AUC index, it can be found that the proposed method is more than 50% compared with the BOW model and 20% higher than the other two deep learning methods. In addition, it can also be found in the Max-Recall index that the proposed method also maintains a higher level than other methods. Due to the environmental changes in the dataset, such as illumination and obstruction of dynamic objects, the proposed method performs better than the traditional BOW model. For CNN and SDA, they directly use the output of the underlying network in the convolutional network. Although they are more accurate than the bag-of-words model, they can easily produce false detection and affect positioning accuracy.

### 4.2 Loop closure detection in a practical environment

In order to further test the effectiveness of the proposed method in a practical environment, we selected sequence images of three complex scenes (sequence numbers 00, 05, and 06) in the KITTI odometry dataset (Geiger et al., 2012) for our experiments; the sequence information is shown in Table 3.

**Table 3:** List of dataset parameters.

**Sequence number of the dataset**	**00**	**05**	**06**
Image size	1,241 × 376	1,241 × 376	1,241 × 376
Number of images	4,540	2,760	1,100
Trajectory length ***(m)***	3,724.187	2,205.576	1,232.876

**Figure 7:**
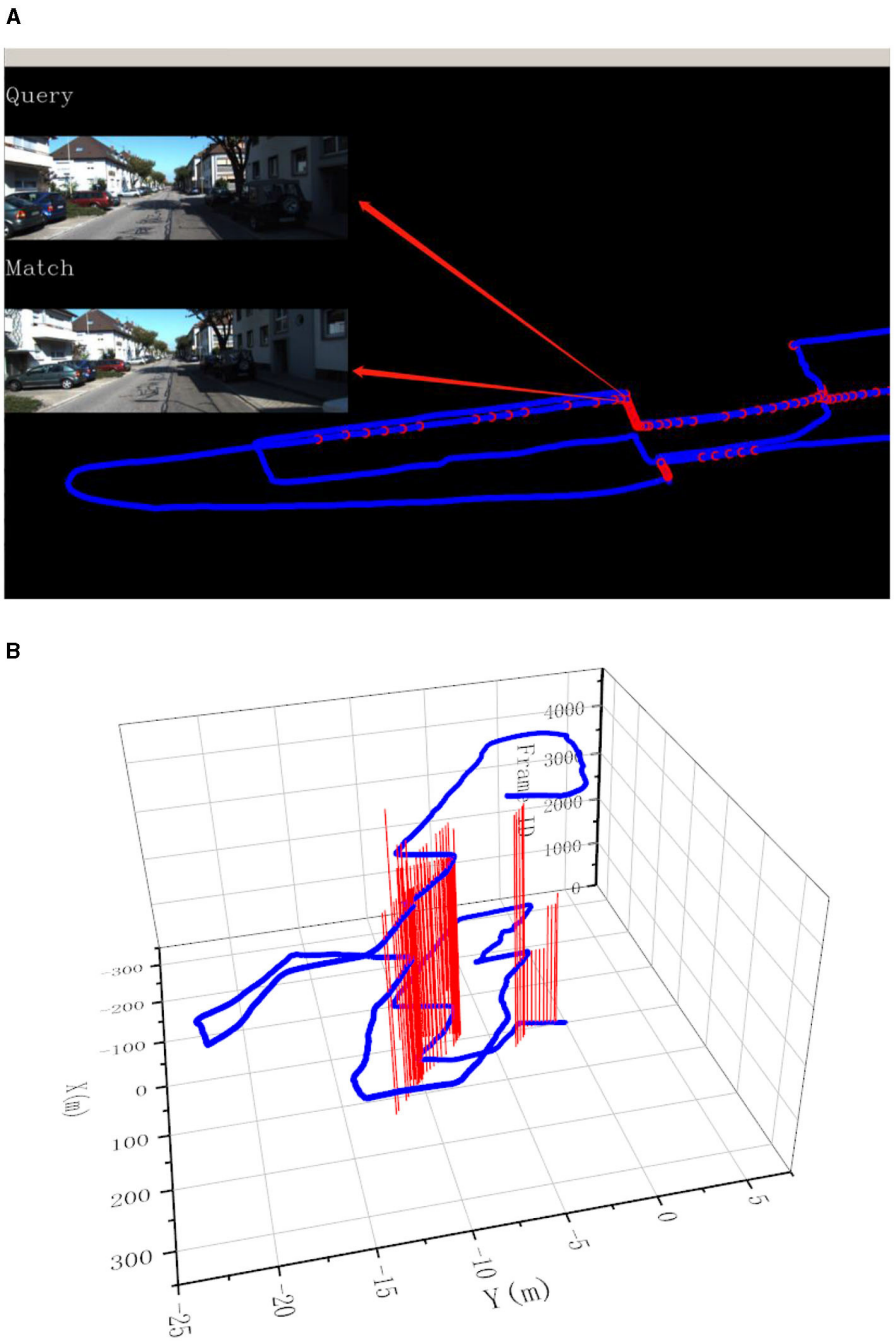
Results of loop closure detection using KITTI-odometry [sequence 00]: **(A)** The screen of online loop closure detection; **(B)** Performance of the proposed method on the practical outdoor dataset.

**Figure 8:**
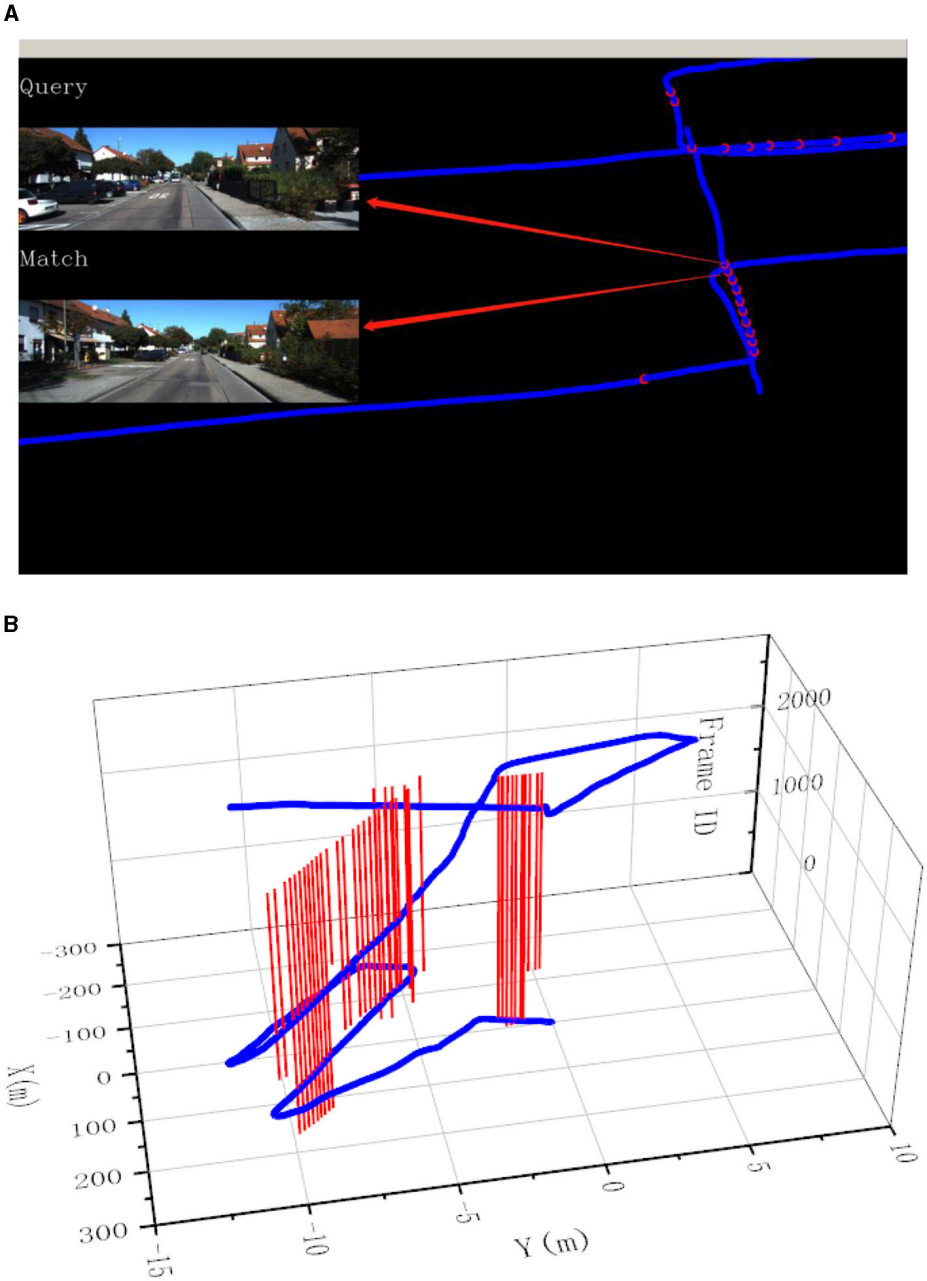
Results of loop closure detection using KITTI-odometry [sequence 05]: **(A)** The screen of online loop closure detection; **(B)** Performance of the proposed method on the practical outdoor dataset.

**Figure 9:**
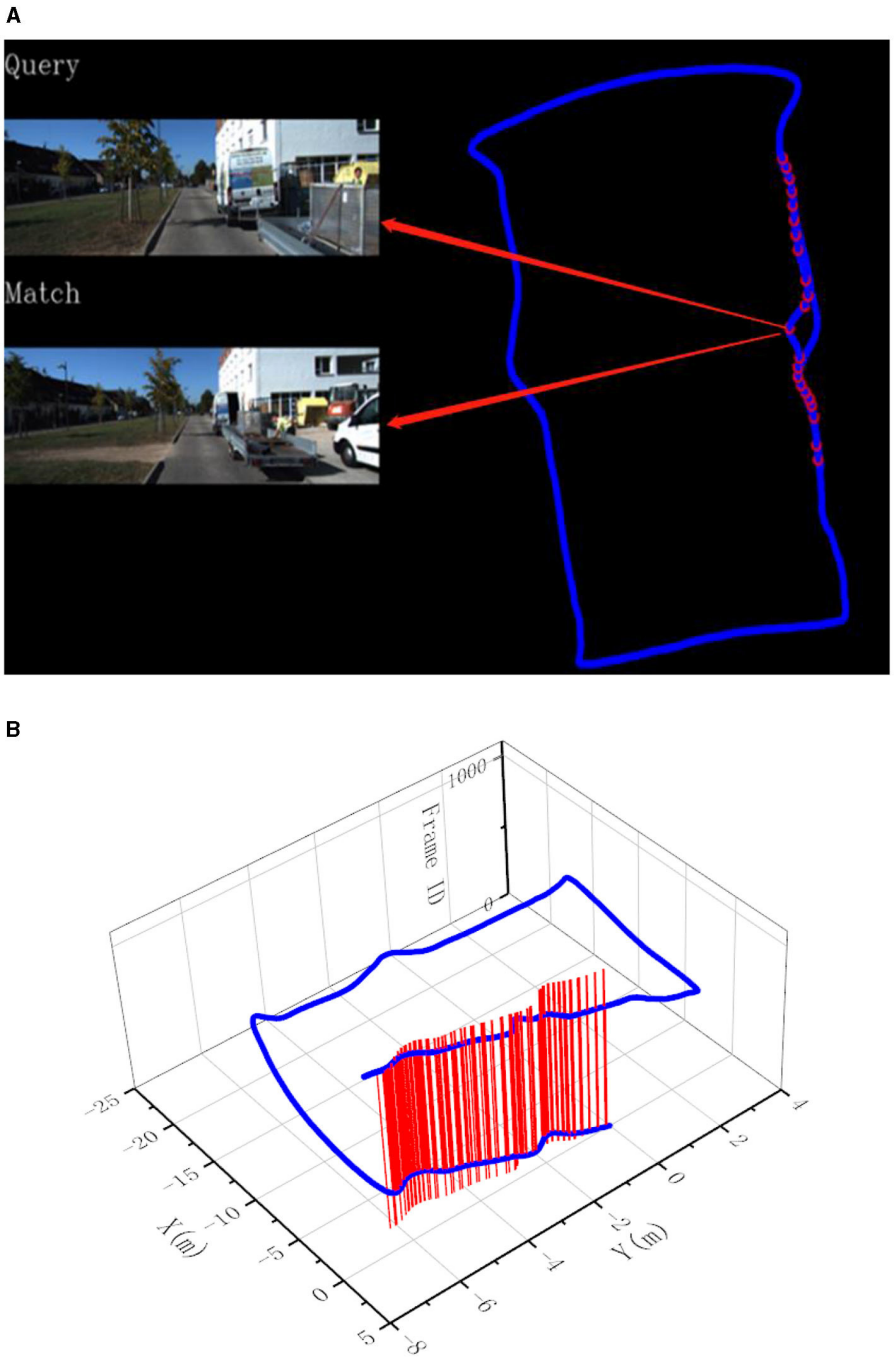
Results of loop closure detection using KITTI-odometry [sequence 06]: **(A)** The screen of online loop closure detection; **(B)** Performance of the proposed method on the practical outdoor dataset.

In this experiment, the image resolution is adjusted to 192 × 256. The final experimental results are presented in Figures 7–9. Part (A) of each figure shows the real-time online detection of the frame, where the red part of the figure represents the image match results with the historical database detected when running to that frame, and the result of the image match is shown on the left. Part (B) of each figure represents the overall trajectory of each sequence. In the figures, the horizontal plane *X*-axis and *Y*-axis represent the distance, and the vertical axis represents the frame index. The red vertical line in the figures represents the detection of a loopback when the trajectory is run to that frame.

**Figure 10:**
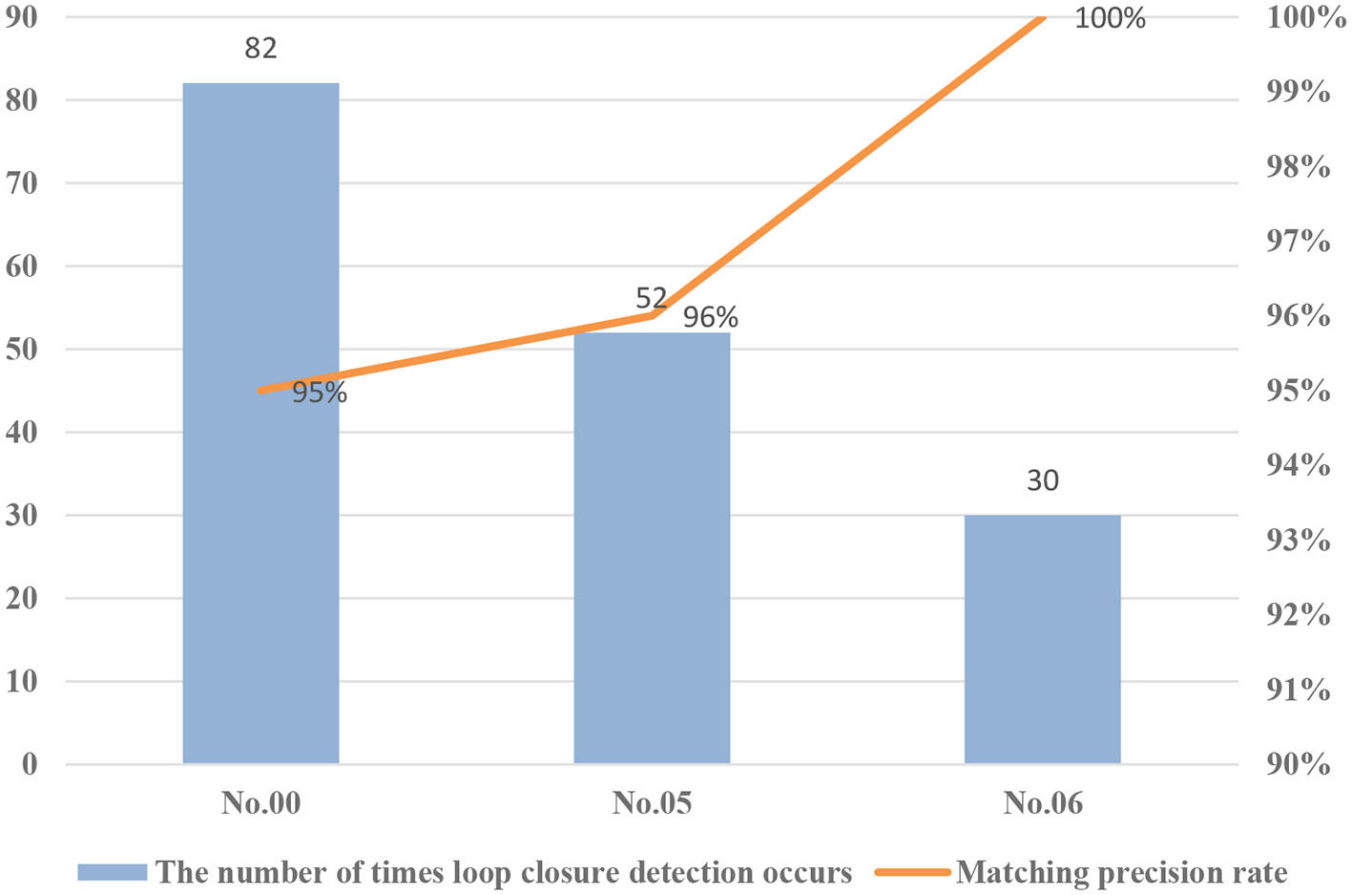
Loop closure detection results under different environments (KITTI dataset of sequence numbers 00, 05, and 06).

For performance evaluation, the number of occurrences of loop closure detection and the accuracy of correctly matching images for each sequence were counted. The test results are shown in Figure 10.

### 4.3 Ablation experimental analysis

As mentioned before, this research paper proposes to incorporate an attention mechanism in the network to filter image features based on feature relevance to improve the performance of the network. This section analyzes the improvement effect of the network from a quantitative point of view and Table 4 shows the results of the experiment. The proposed method is trained on the COCO dataset (Caesar et al., 2018). It should be noted that when β = 1, it is equivalent to the original Kullback-Leibler divergence loss. When increasing the value β, we can see that the Kullback-Leibler divergence loss has a significant decrease, which indicates that the encoder maps the input distribution closer to the desired normal distribution, and the model has a better disentangling ability. Furthermore, with the addition of the attention mechanism, the model improves by 4.9% in recall and accuracy compared to not adding the module.

**Table 4:** Ablation experiments on different modules of the network.

**Method**	**β = 1**	**β = 250**	**SENet**	**Metrics**	
				**KLD loss**	**AUC**
Ours	**√**			1,575.23	0.7723
Ours		**√**		42.73	0.8042
Ours		**√**	**√**	**44.68**	**0.8453**

The bold values represent the final values obtained by the model after improving the loss function and adding the attention mechanism.

## 5 Conclusions

In this research paper, a loop closure detection method based on a variational autoencoder is documented, which uses a neural network to learn the representation of the image from the original image to replace the traditional handicraft features. We incorporate an attention mechanism in the coding layer of the neural network, which can automatically obtain the feature weight of each feature channel, and then improve the performance of the network in extracting image features by improving the features that are useful for the current task and suppressing the useless ones according to this feature weight. At the same time, the loss function of the variational autoencoder (VAE) is improved. By adding a hyperparameter β to the second KL divergence term of the loss function, the VAE shows better disentanglement ability and improves the performance and convergence of the network. Experiments on the Campus Loop dataset show that the proposed method can maintain high accuracy at a high recall rate. In addition, experiments on the datasets for three different scenarios indicate that the method is robust to environmental changes, and can maintain high accuracy even in the presence of viewing angle changes and object occlusions. Our future work will consider lightweight design and modification of the method to adapt it to practical high-speed scenarios.

## Data availability statement

The datasets presented in this article are not readily available because the datasets used or analyzed during the current study are available from the corresponding author on reasonable request. Requests to access the datasets should be directed at: SS, shbsong_skd@sdust.edu.cn.

## Author contributions

SS: Conceptualization, Funding acquisition, Writing—review & editing. FY: Conceptualization, Methodology, Validation, Writing—original draft, Writing—review & editing. XJ: Validation, Writing—review & editing. JZ: Software, Writing—review & editing. WC: Validation, Writing—review & editing. XF: Visualization, Writing—review & editing.

## References

[B1] Arandjelovic R. Gronat P. Torii A. Pajdha T. Sivic J. (2016). NetVLAD: CNN architecture for weakly supervised place recognition, in Proceedings of the IEEE Conference on Computer Vision and Pattern Recognition (IEEE: Las Vegas, NV), 5297–5307. 10.1109/CVPR.2016.572 28622667

[B2] Arshad S. Kim G.-W. (2021). Role of deep learning in loop closure detection for visual and lidar slam: a survey. Sensors 21, 1243. 10.3390/s21041243 33578695 PMC7916334

[B3] Bay H. Tuytelaars T. Gool L. V. (2006). Surf: speeded up robust features. Lect. Notes Comput. Sci. 3951, 404–417. 10.1007/11744023_32

[B4] Bengio Y. Courville A. Vincent P. (2013). Representation learning: a review and new perspectives. Trans. Pattern Anal. Mach. Intell. 35, 1798–1828. 10.1109/TPAMI.2013.50 23787338

[B5] Caesar H. Uijlings J. Ferrari V. (2018). COCO-stuff: thing and stuff classes in context, in 2018 IEEE Conference on Computer Vision and Pattern Recognition (CVPR) (Salt Lake City, UT: IEEE). 10.1109/CVPR.2018.00132

[B6] Calonder M. Lepetit V. Strecha C. Fua P. (2010). Brief: binary robust independent elementary features, in Computer Vision–ECCV 2010: 11th European Conference on Computer Vision, Heraklion, Crete, Greece, September 5-11, 2010 Proceedings, Part IV 11 (Berlin: Springer), 778–792. 10.1007/978-3-642-15561-1_56

[B7] Cummins M. Newman P. (2008). FAB-MAP: probabilistic localization and mapping in the space of appearance. Int. J. Rob. Res. 27, 647–665. 10.1177/0278364908090961

[B8] Farrukh F. U. D. Zhang W. Zhang C. Wang Z. Jiang H. (2022). FPSNET: an architecture for neural-network-based feature point extraction for SLAM. Electronics 11, 4168. 10.3390/electronics11244168

[B9] Favorskaya M. N. (2023). Deep learning for visual SLAM: the state-of-the-art and future trends. Electronics 12, 2006. 10.3390/electronics12092006

[B10] Filliat D. (2007). A visual bag of words method for interactive qualitative localization and mapping, in International Conference on Robotics and Automation (Rome: IEEE), 3921–3926. 10.1109/ROBOT.2007.364080

[B11] Gálvez-López D. Tardis J. D. (2012). Bags of binary words for fast place recognition in image sequences. IEEE Trans. Robot. 28, 1188–1197. 10.1109/TRO.2012.2197158

[B12] Gao X. Zhang T. (2017). Unsupervised learning to detect loops using deep neural networks for visual SLAM system. Auton. Robots. 41, 1–18. 10.1007/s10514-015-9516-2

[B13] Garcia-Fidalgo E. Ortiz A. (2018). IBoW-LCD: an appearance-based loop-closure detection approach using incremental bags of binary words. IEEE Robot. Autom. Lett. 3, 3051–3057. 10.1109/LRA.2018.2849609

[B14] Garg S. Suenderhauf N. Milford M. (2018). Lost? appearance-invariant place recognition for opposite viewpoints using visual semantics. arXiv. [Preprint]. 10.48550/arXiv.1804.05526

[B15] Geiger A. Lenz P. Urtasun R. (2012). Are we ready for autonomous driving? The kitti vision benchmark suite, in 2012 IEEE Conference on Computer Vision and Pattern Recognition (Providence, RI: IEEE), 3354–3361. 10.1109/CVPR.2012.6248074

[B16] Hou Y. Zhang H. Zhou S. (2015). Convolutional neural network-based image representation for visual loop closure detection, in IEEE International Conference on Information and Automation (Lijiang: IEEE), 2238–2245. 10.1109/ICInfA.2015.7279659

[B17] Hu J. Shen L. Sun G. (2018). Squeeze-and-excitation networks, in Proceedings of the IEEE Conference on Computer Vision and Pattern Recognition (Salt Lake City, UT: IEEE), 7132–7141. 10.1109/CVPR.2018.00745

[B18] Li S. Zhang T. Gao X. Wang D. Xian Y. (2019). Semi-direct monocular visual and visual-inertial SLAM with loop closure detection. Robot. Auton. Syst. 112, 201–210. 10.1016/j.robot.2018.11.009

[B19] Liu K. Cao M. (2023). Dlc-slam: a robust lidar-slam system with learning-based denoising and loop closure. IEEE/ASME Trans. Mechatron. 28, 2876–2884. 10.1109/TMECH.2023.3253715

[B20] Liu K. Gao Z. Lin F. Chen B. M. (2020). FG-Net: fast large-scale LiDAR point clouds understanding network leveraging correlated feature mining and geometric-aware modelling. arXiv. [Preprint]. 10.48550/arXiv.2012.09439

[B21] Lowe D. G. (2004). Distinctive image features from scale-invariant keypoints. Int. J. Comput. Vis. 60, 91–110. 10.1023/B:VISI.0000029664.99615.94

[B22] Malkov Y. A. Yashunin D. A. (2018). Efficient and robust approximate nearest neighbor search using hierarchical navigable small worldgraphs. IEEE Trans. Pattern Anal. Mach. Intell. 42, 824–836. 10.1109/TPAMI.2018.2889473 30602420

[B23] Memon A. R. Wang H. Hussain A. (2020). Loop closure detection using supervised and unsupervised deep neural networks for monocular SLAM systems. Rob. Auton. Syst. 126, 103470. 10.1016/j.robot.2020.103470

[B24] Merrill N. Huang G. (2018). Lightweight unsupervised deep loop closure, in Proc. of Robotics: Science and Systems (RSS) (Pittsburgh, PA). 10.15607/RSS.2018.XIV.032

[B25] Mur-Artal R. Martinez Montiel J. M. Tardos J. D. (2015). ORB-SLAM: a versatile and accurate monocular SLAM system. IEEE Trans. Robot. 31, 1147–1163. 10.1109/TRO.2015.2463671

[B26] Mur-Artal R. Tardós J. D. (2017). Orb-slam2: an open-source slam system for monocular, stereo, and RGB-D cameras. IEEE Trans. Robot. 33, 1255–1262. 10.1109/TRO.2017.2705103

[B27] Osman H. Darwish N. Bayoumi A. (2023). PlaceNet: a multi-scale semantic-aware model for visual loop closure detection. Eng. Appl. Artif. Intell. 119, 105797. 10.1016/j.engappai.2022.105797

[B28] Qin H. Huang M. Cao J. Zhang X. (2018). Loop closure detection in SLAM by combining visual CNN features and submaps, in Proceedings of the 4th International Conference on Control, Automation and Robotics, ICCAR, Auckland, New Zealand, 20–23 April (Auckland: IEEE), 426–430. 10.1109/ICCAR.2018.8384713

[B29] Safron A. Çatal O. Verbelen T. (2022). Generalized simultaneous localization and mapping (G-SLAM) as unification framework for natural and artificial intelligences: towards reverse engineering the hippocampal/entorhinal system and principles of high-level cognition. Front. Syst. Neurosci. 16, 787659. 10.3389/fnsys.2022.787659 36246500 PMC9563348

[B30] Schönberger J. L. Pollefeys M. Geiger A. Sattler T. (2018). Semantic visual localization, in Proceedings of the IEEE Conference on Computer Vision and Pattern Recognition (Salt Lake City, UT: IEEE), 6896–6906. 10.1109/CVPR.2018.00721

[B31] Sikka H. Zhong W. Yin J. Pehlevan C. (2019). A closer look at disentangling in β-VAE, in 2019 53rd Asilomar Conference on Signals, Systems, and Computers (Pacific Grove, CA: IEEE), 888–895. 10.1109/IEEECONF44664.2019.9048921

[B32] Wang S. Lv X. Liu X. Ye D. (2020). Compressed holistic convnet representations for detecting loop closures in dynamic environments. IEEE Access 8, 60552–60574. 10.1109/ACCESS.2020.2982228

[B33] Zhang K. Ma J. Jiang J. (2022). Loop closure detection with reweighting NetVLAD and local motion and structure consensus. IEEE/CAA J. Autom. Sin. 9, 1087–1090. 10.1109/JAS.2022.105635

[B34] Zhang X. Su Y. Zhu X. (2017). Loop closure detection for visual SLAM systems using convolutional neural network, in 23rd International Conference on Automation and Computing (ICAC) (Huddersfield: IEEE), 1–6. 10.23919/IConAC.2017.8082072

